# Hypomethylated domain-enriched DNA motifs prepattern the accessible nucleosome organization in teleosts

**DOI:** 10.1186/s13072-017-0152-2

**Published:** 2017-09-20

**Authors:** Ryohei Nakamura, Ayako Uno, Masahiko Kumagai, Shinichi Morishita, Hiroyuki Takeda

**Affiliations:** 1 Department of Biological Sciences, Graduate School of Science, The University of Tokyo, 7-3-1 Hongo, Bunkyo-ku, Tokyo, 113-0033 Japan; 2 Department of Computational Biology and Medical Sciences, Graduate School of Frontier Sciences, The University of Tokyo, 5-1-5 Kashiwanoha, Kashiwa, 277-8562 Japan

**Keywords:** Nucleosome positioning, DNA methylation, DNA sequence, Vertebrate

## Abstract

**Background:**

Gene promoters in vertebrate genomes show distinct chromatin features such as stably positioned nucleosome array and DNA hypomethylation. The nucleosomes are known to have certain sequence preferences, and the prediction of nucleosome positioning from DNA sequence has been successful in some organisms such as yeast. However, at gene promoters where nucleosomes are much more stably positioned than in other regions, the sequence-based model has failed to work well, and sequence-independent mechanisms have been proposed.

**Results:**

Using DNase I-seq in medaka embryos, we demonstrated that hypomethylated domains (HMDs) specifically possess accessible nucleosome organization with longer linkers, and we reassessed the DNA sequence preference for nucleosome positioning in these specific regions. Remarkably, we found with a supervised machine learning algorithm, *k*-mer SVM, that nucleosome positioning in HMDs is accurately predictable from DNA sequence alone. Specific short sequences (6-mers) that contribute to the prediction are specifically enriched in HMDs and distribute periodically with approximately 200-bp intervals which prepattern the position of accessible linkers. Surprisingly, the sequence preference of the nucleosome and linker in HMDs is opposite from that reported previously. Furthermore, the periodicity of specific motifs at hypomethylated promoters was conserved in zebrafish.

**Conclusion:**

This study reveals strong link between nucleosome positioning and DNA sequence at vertebrate promoters, and we propose hypomethylated DNA-specific regulation of nucleosome positioning.

**Electronic supplementary material:**

The online version of this article (doi:10.1186/s13072-017-0152-2) contains supplementary material, which is available to authorized users.

## Background

Eukaryotic genomes are organized into chromatin, a DNA–protein complex, together with epigenetic information such as nucleosome position, histone modification, and DNA methylation. A nucleosome is a basic packaging unit of chromatin consisting of 147 base pairs (bp) DNA wrapped around a histone octamer [1]. Positioning of nucleosomes affects accessibility of regulatory proteins to DNA and thereby influences gene transcription [2]. Histone modification and DNA methylation also play critical roles in transcriptional regulation, and regulatory DNA regions such as promoters and enhancers are characterized by specific histone modifications, DNA hypomethylation, and accessible nucleosome organization [3–7].

Using next generation sequencing techniques, many studies have attempted to identify the basic principle for nucleosome positioning and have found that nucleosomes have DNA sequence preference. For example, nucleosome formation tends to occur at 10-bp periodic repeat of AT/TA dinucleotides and also GC-rich sequences, whereas poly(dA:dT) sequences tend to evict nucleosomes and thus reside in the linker region [8, 9]. Indeed, a periodic DNA sequence pattern associated with nucleosome has been found in genomes [10]. Furthermore, genome-wide nucleosome mapping in yeast and *C. elegans* revealed that the position of nucleosomes on the genome is accurately predictable from DNA sequences [11], suggesting a certain dependency of nucleosome positioning on local DNA sequences in these organisms. However, in more complex organisms such as vertebrates the prediction from DNA sequence has not been successful [12, 13]. These facts suggest that the sequence dependency of nucleosome positioning varies among species.

The promoter region is unique in the genome, because nucleosomes at gene promoters are known to be stably positioned and strongly phased, which is one of the widely conserved features of nucleosome organization in eukaryotes including vertebrates [13–18]. In spite of these characteristics, the prediction of nucleosome positions in promoter regions from DNA sequence has not been successful even in yeast [11–13], suggesting that nucleosome positioning in promoter regions relies on other rules. Indeed, a transacting factor-mediated mechanism has been proposed in the promoter region [8, 18, 19]. One exception reported so far is tetrahymena, in which nucleosome positioning downstream of TSSs coincides significantly with GC content [14]. However, the logic underlying nucleosome positioning at promoters remains elusive for other organisms.

In vertebrates, the majority of the genome is maintained methylated, and hypomethylated domains (HMDs) are predominantly found in the region around gene promoters [20]. HMDs are mostly enriched with specific histone modifications such as H3K4me and required for gene transcription [21–23]. Recent studies have utilized a supervised machine learning algorithm, the *k*-mer support vector machine (SVM), and showed that HMDs can be accurately predicted from DNA sequence alone in *Xenopus* embryos and that these HMD regions are highly enriched with specific *k*-mers [24]. Importantly, the link between epigenetic modifications and nucleosome positioning has been also reported [13, 18, 25, 26], and epigenetic modification could be one of the key factors which affect nucleosome positioning. Given that majority of gene promoters are overlapped with HMDs, vertebrate promoter regions are distinct from the rest of the genomic regions in terms of both epigenetic modification and DNA sequence composition. Thus, distinct mechanism for nucleosome positioning might exist in promoter regions.

Here, we investigated the nucleosome organization and the contribution of DNA sequences to nucleosome positioning in HMDs using the medaka (Japanese killifish). We found that the nucleosome linkers in HMDs are specifically accessible, and their positions can be precisely mapped using DNase I-seq in medaka embryos. The nucleosome linkers in HMDs are longer than typical ones in the methylated medaka genome, and the average nucleosome spacing changes sharply at the boundary of HMDs (200 bp in HMDs and 180 bp in methylated regions). Unlike the previous notion, the nucleosome positioning within HMDs was found to be highly predictable from DNA sequence using *k*-mer SVM, suggesting that nucleosome positioning in HMDs depends significantly on its proximal linker sequence. Surprisingly, this sequence feature was opposite from the previously reported global sequence preference of nucleosome in yeast. Finally, the specific sequence occurrence in hypomethylated linkers was also observed in zebrafish, a distantly related teleost species. Taken together, we propose a novel epigenetic modification-dependent and sequence-based rule for nucleosome positioning at teleost promoters.

## Results

### HMD have specific nucleosome organization

**Fig. 1:**
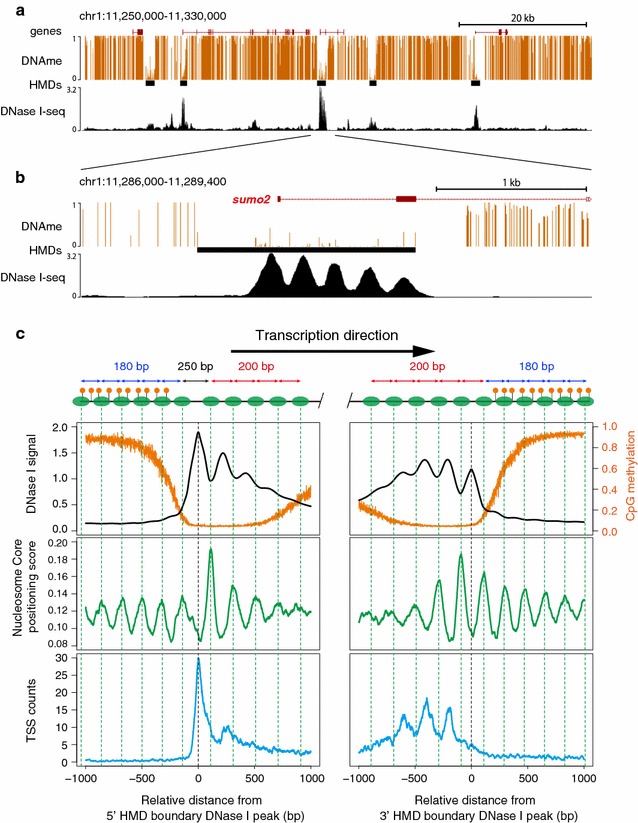
DNase I-seq detects accessible nucleosome linkers within HMDs. **a** A representative genome browser view of DNA methylation, HMDs, and DNase I-seq pattern (signals per million reads) in medaka blastula embryos. Vertical line height of DNA methylation track indicates the ratio of methylated CpG. Black boxes represent HMDs. **b** A close-up view of single HMDs in (**a**). **c** Average profiles of DNase I-seq signal, DNA methylation, nucleosome core, and TSS counts around the accessible nucleosome linkers at the HMD boundaries. Vertical green dashed lines indicate the position of nucleosome core estimated from MNase-seq data. The top schema shows the position of nucleosomes (green ovals) and methylated CpGs (orange circle)

We previously reported 15,145 HMDs containing at least 10 continuous low-methylated (methylation rate < 0.4) CpGs in the genome of medaka blastula embryos, and the majority (69%) of the HMDs are found in gene promoter regions [23]. To examine the nucleosome organization within the HMD, we made a map of accessible chromatin in the medaka blastula genome using DNase I-seq. DNase I preferentially digests accessible DNA, such as nucleosome linkers or nucleosome-depleted regions [27, 28]. By deep sequencing, 323 million reads generated by DNase I digestions were mapped to the medaka reference genome and 36,375 DNase I hypersensitive sites (DHSs) were identified using MACS2 software [29] by searching regions with significant enrichment (FDR < 0.1%, fold enrichment > 5) of DNase I cleavage. As expected, DHSs were highly enriched in HMDs (Fig. 1a); 84.8% of HMDs contained at least one DHS, and 40.7% of DHSs are found in the HMD which constitutes only 3% of the blastula genome. Notably, the DNase I-seq pattern in HMDs showed the clear periodic pattern (Fig. 1b), suggesting that the DNase I cleavage pattern in the medaka blastula genome represents arrays of long and accessible nucleosome linkers that specifically exist in HMDs.

To examine if the periodic DNase I-seq pattern reflects the array of nucleosome linkers in HMDs and if the nucleosome linker length is specifically longer in HMDs than in methylated regions, we compared the periodic DNase I cleavage pattern with our previous MNase-seq data in medaka blastula embryos [30]. To clarify the difference in nucleosome organization between HMDs and methylated regions, the DNase I-seq peak summits that reside at the most end of the HMD were designated as the base position. As nucleosomes are known to show strong phasing especially downstream of TSSs [2, 30], we wanted to distinguish the change in nucleosome phasing at HMD boundaries from TSS-dependent phasing. To this end, we oriented each HMD boundary by the direction of transcriptions from its nearest TSS (i.e., if the direction of the transcription was from the methylated side toward hypomethylated side, the boundary was classified as 5′ boundary, and 3′ boundary in the opposite case). First, we confirmed that the periodic pattern of DNase I-seq is inversely correlated with the nucleosome position estimated from the MNase-seq data (Fig. 1c; top and middle). In some cases, MNase-seq data could be affected by nonhistone DNA binding proteins [31]. However, we confirmed that the periodic patterns of DNase I-seq and MNase-seq are consistent with our previously published histone ChIP-seq pattern [23] (Additional file 1). These results indicate that the periodic DNase I-seq signals indeed reflected the nucleosome linkers in HMDs. Next, we observed that TSSs were most frequently found at the accessible linker located at the 5′ edge of HMDs, i.e., on the base position (Fig. 1c; bottom left). On the other hand, at 3′ boundary of HMDs, several peaks of TSS counts appear at linkers upstream of the base position (Fig. 1c; right bottom), probably reflecting TSSs at the 5′ boundary in short HMDs. Surprisingly, we found that the average spacing of nucleosomes changed clearly at the HMD boundary irrespective of the direction of transcription; in the methylated region, nucleosomes showed approximately 180-bp spacing, but in HMDs, the spacing was approximately 200 bp (Fig. 1c; Additional file 2). The spacing at 5′ HMD boundary was especially long (~250 bp), which is reminiscent of the fact that nucleosome-depleted region (NDR) exists at TSSs [2, 18]. Taken together, HMDs have distinct nucleosome organization, and our DNase I-seq data preferentially detect nucleosome linkers in HMDs that are longer (~200 bp) than typical linkers (~180 bp) in medaka embryos.

### Prediction of nucleosome positioning by *k*-mer SVM

**Fig. 2:**
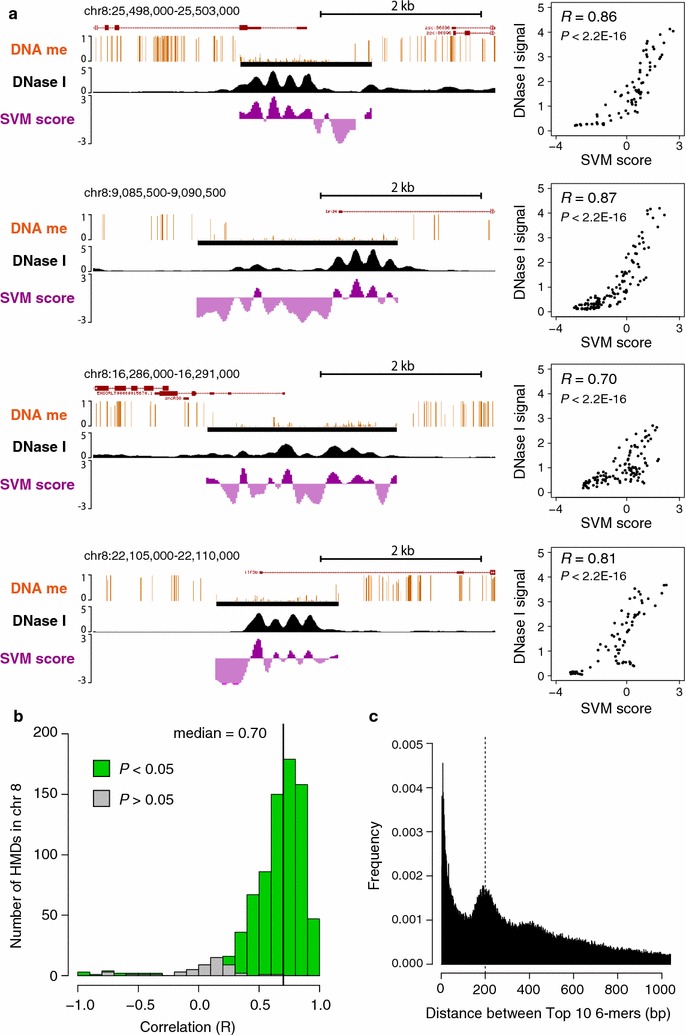
Nucleosome positioning in HMDs is predictable by *k*-mer SVM. **a** Examples of prediction of nucleosome linkers (DNase I accessible regions) by *k*-mer SVM in HMDs on chromosome 8. Dark purple indicates the score higher than 0, light purple, lower than 0. Pearson’s correlation and its *P* value between DNase I signal and SVM score for every 20 bp along the HMD are shown on the right. **b** A histogram of correlations for all HMDs on chromosome 8. Green and gray boxes represent the number of HMDs with and without significant correlation (*P* < 0.05), respectively. **c** A histogram of distances between top 10 SVM_DNaseI_-weight 6-mers within HMDs. Distances shorter than 3 bp were excluded from the histogram

Since we precisely mapped the position of nucleosome linkers in each HMD, we then asked if specific DNA sequences can be correlated with the positioning of accessible linkers. *k*-mer-based DNA sequence analyses have been utilized to identify specific DNA elements [32]. We applied *k*-mer SVM, which finds a decision boundary that distinguishes the two sets of sequence data based on the frequency of all possible *k*-mers [33]. To discriminate linker sequences from nucleosome core sequences in HMDs, we extracted 100-bp sequences from DNase I peak summits in HMDs as positive (linker) data, and 100-bp sequences from the center regions between the two adjacent DNase I peak summits within HMDs for negative (core) data. Sequences on chromosome 8 were separated and used as test data, and the remaining sequences were used as training data. The performance of the *k*-mer SVM differed slightly between different *k*-mer length (*k* = 2, 3, 4, 5, 6, 7, 8), and we chose to use 6-mers for the further analyses, as this length produced high performance with minimized overfitting (Additional file 3). We refer to this trained SVM as SVM_DNaseI_, as its purpose is to predict the DNase I-seq peaks (i.e., linkers) in HMDs. If nucleosome positioning in HMDs depends on specific DNA motifs, it should be predicted from DNA sequence. We calculated the SVM score for every 20 bp within HMDs on chromosome 8 and compared with DNase I-seq signal strength. Remarkably, SVM_DNaseI_ accurately predicted the DNase I pattern in HMDs (Fig. 2a left), and the correlation between the SVM_DNaseI_ score and actual DNase I-seq signal was significantly strong in each HMD (Fig. 2a right). This strong correlation was observed for the majority of HMDs on chromosome 8 (Fig. 2b). DNase I has been reported to have sequence preference [34–36], and thus the trained SVM might have been affected by this cleavage bias. In order to confirm that the SVM_DNaseI_ actually predicts nucleosome positioning, we performed ATAC-seq, an alternative method to map chromatin accessibility by Tn5 transposase [37], and compared with the SVM score. We found that the SVM_DNaseI_ score also showed significant correlation with ATAC-seq signal (Additional file 4). These results revealed that nucleosome positioning in HMDs is predictable from 6-mer distributions, suggesting that a sequence-based rule dominates in HMDs.

### Specific 6-mers periodically distribute with 200-bp intervals in the linker regions of HMDs

The SVM outputs a weight for each *k*-mer which corresponds to the degree it contributes to the prediction [33] (Additional file 5). In this case, 6-mers with large positive weights were most frequently found in linker sequences, whereas those with large negative weights tended to be excluded from linkers but present in nucleosome core sequences. We noticed that the top positive 6-mers have larger absolute weights than top negative ones (Additional file 5), suggesting that a few number of specific 6-mers in linkers have strong contribution to nucleosome positioning. To test whether the high SVM-weight 6-mers appear periodically in a single HMD, we examined the distances between every pairs of top 10 high-weight 6-mers of SVM_DNaseI_ within HMDs. The histogram of all distances between the top 6-mer pairs showed clear enrichment at 200 bp (Fig. 2c), indicating that those top 6-mers tend to distribute with approximately 200-bp intervals within a HMD.

**Fig. 3:**
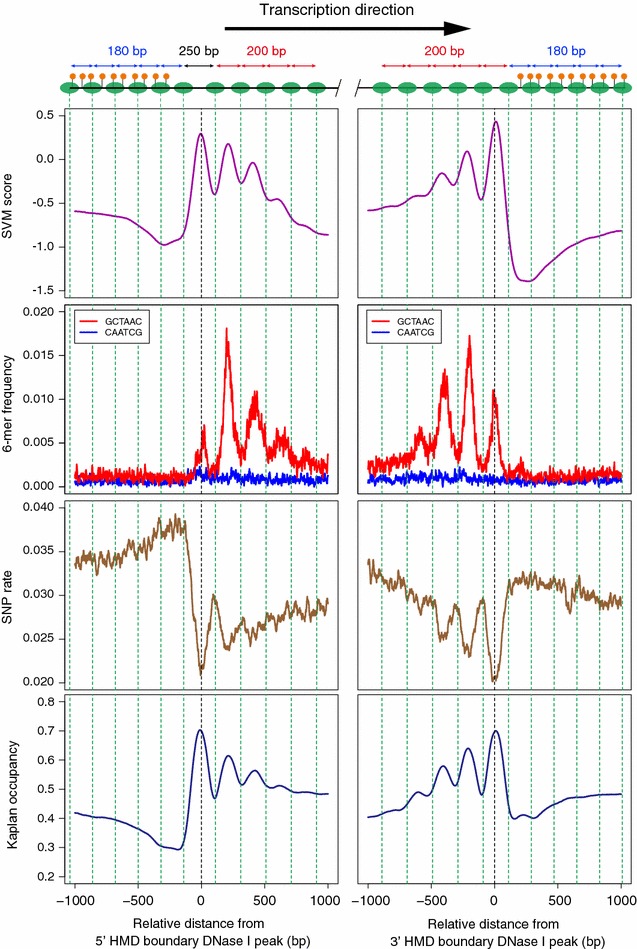
Specific DNA 6-mers are enriched in accessible linkers. Average profiles of the SVM score, distribution frequency of 6-mers with highest SVM-weight (GCTAAC) and its reverse sequence (CAATCG), SNP rate, and Kaplan occupancy around the HMD boundaries. Vertical green dashed lines indicate the position of nucleosome core, and the top schema shows the position of nucleosomes (green ovals) and methylated CpGs (orange circle) (same as Fig. 1c)

We then examined the pattern of the SVM score around the HMD boundary and confirmed that the SVM score shows a periodical pattern with high levels at nucleosome linker regions specifically in HMDs (Fig. 3), suggesting that specific DNA motifs strongly contribute to the nucleosome positioning in HMDs. It is known that nucleosomes have specific sequence preference, poly(dA:dT) sequences for linkers and relatively GC-rich for nucleosome cores [8, 9]. Thus, the enrichment of specific 6-mers at the nucleosome linkers could be the result of distinct base compositions. To test this idea, we examined the distribution pattern of 6-mers with the highest SVM_DNaseI_-weight (GCTAAC) and its reverse sequence (CAATCG) which is not reverse-complement but has the same base composition to highlight the importance of the base ordering in the motif. The highest SVM_DNaseI_-weight 6-mer showed the clear periodic distribution pattern that is consistent with the position of linkers in HMDs, whereas the reverse sequence did not show such pattern (Fig. 3). These results suggest that specific DNA motifs, but not simple base composition, contribute to the formation of accessible nucleosome linkers. Furthermore, the SNP rate between the two closely related medaka species, Hd-rR and HNI [38–41], also showed a periodic pattern, indicating that nucleosome linker regions are highly conserved in HMDs (Fig. 3). This further suggests the importance of linker sequences. The eviction of nucleosomes from specific 6-mers could be caused by the binding of certain proteins to those specific sequences. However, the majority of high SVM-weight 6-mers do not show any similarity to known TF binding motifs (Additional file 6). Thus, intrinsic preference of the specific 6-mers for nucleosome linkers may exist in HMDs.

Previously, the global sequence preference of nucleosome has been proposed to predict in vivo genome-wide nucleosome positioning in yeast and *C. elegans* [11]. However, this model has limited performance when applied to human and zebrafish genomes [12, 13]. To test whether this model can be applied to the nucleosome positioning in HMDs, we calculated the Kaplan occupancy (expected nucleosome occupancy) around HMD boundaries. As shown in Fig. 3, the Kaplan occupancy showed the clear periodic pattern similar to that of the SVM score. This result was surprising, because the Kaplan occupancy is known to predict the nucleosome core position, but the SVM score correlates with the linker region in HMDs. Thus, nucleosomes in medaka HMDs have the sequence preference opposite to the global tendency in yeast.

### Linker-specific 6-mers distribute preferentially in HMDs

**Fig. 4:**
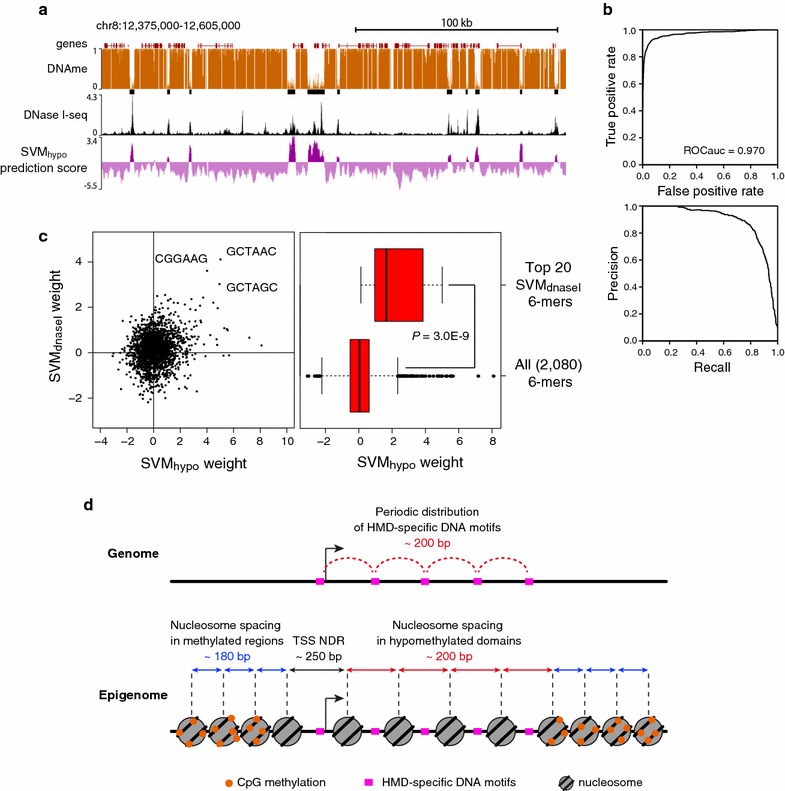
Specific 6-mers enrichment in linkers are unique to HMDs. **a** An example of prediction of HMDs by SVM_hypo_. The DNA methylation ratio, HMDs at the medaka blastula stage, and SVM prediction score (dark purple indicates a score higher than −1, light purple, lower than −1) are shown. **b** Performance of classification of HMD versus methylated sequences on chromosome 8 by SVM_hypo_. ROC curve and the area under the ROC curve (ROCauc) (top), and precision–recall curve (bottom) are shown. **c** Comparison of SVM-weights between SVM_hypo_ and SVM_DNaseI_ for all 6-mers (left), and boxplots shows the difference of SVM_hypo_-weights between the top 20 SVM_DNaseI_ 6-mers and all 6-mers. *P* value was calculated using non-paired Wilcoxon test. **d** A schematic of nucleosome positioning and specific DNA motif distribution in presumptive HMDs

We reasoned that the specific localization of high SVM-weight 6-mers is only observed in HMDs (Fig. 2) but not in the methylated region. To examine whether those 6-mers are actually enriched in HMDs, we trained *k*-mer SVM to discriminate HMD sequences from randomly selected methylated sequences and compared the contribution to the prediction of each 6-mers between HMDs and nucleosome linkers. We refer to this new trained SVM as SVM_hypo_, as it is to predict the HMD. The performance of SVM_hypo_ was tested on HMDs and methylated sequences from chromosome 8, and the prediction quality was measured by calculating the area under the ROC curve (ROCauc). Consistent with the previous study [24], the SVM_hypo_ was able to distinguish HMD sequences from methylated sequences with high accuracy (Fig. 4a, b). Furthermore, we also measured ‘precision and recall,’ as it is a more reliable measure when positive and negative datasets are of unequal size. The precision–recall curve revealed that the SVM_hypo_ can distinguish HMD sequences from a 10× excess of methylated sequences (Fig. 4b). These results demonstrate that HMDs in blastula embryos are specifically enriched with a certain set of 6-mers. Intriguingly, the comparison between the SVM-weight of each 6-mer by SVM_hypo_ and SVM_DNaseI_ demonstrated that high SVM_DNaseI_-weight 6-mers tended to have high SVM_hypo_ weight (i.e., the top 20 SVM_DNaseI_-weight 6-mers had significantly high SVM_hypo_-weights) (Fig. 4c). Thus, the 6-mers that contribute to the prediction of nucleosome linkers are preferentially distributed in HMDs and much less frequently present in methylated regions. This suggests that the sequence-based rule we propose is specific to the HMD, but should not be applicable to the methylated genomic region which constitutes the majority of the genome.

Taken together, accessible nucleosome organization in HMDs might uniquely depend on DNA sequence, which is directed by specific short sequences preferentially distributed with approximately 200-bp intervals in HMDs, longer than those in methylated regions (~180 bp) (Fig. 4d).

### Similar sequence preference of nucleosome positioning in zebrafish HMDs

**Fig. 5:**
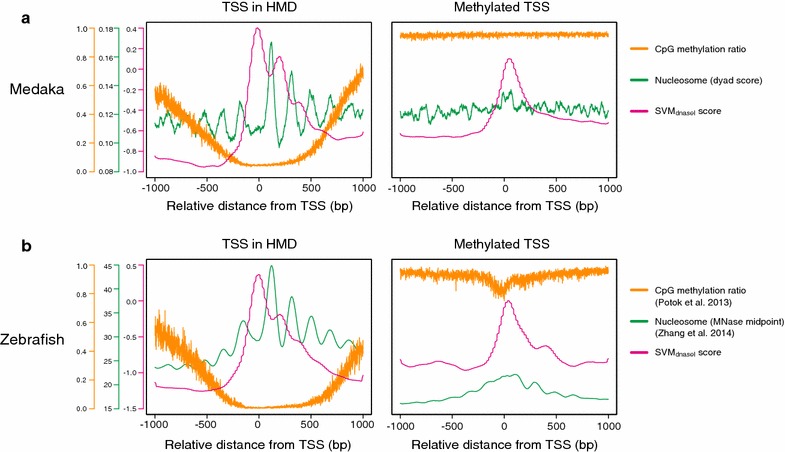
HMD-specific DNA sequence preference for the nucleosome linker is conserved among teleosts. **a**, **b** Average DNA methylation, nucleosome position, and SVM_DNaseI_ score around TSSs in HMDs (left) and methylated regions (right). For both medaka (**a**) and zebrafish (**b**), SVM_DNaseI_ score was calculated using SVM trained by medaka data sets

Finally, we tested whether the unique sequence preference of nucleosomes in HMDs also exists in other vertebrate species. We examined the sequence features of nucleosome core and linker regions in zebrafish by investigating the SVM_DNaseI_ score, together with the published data of methylome [42] and MNase-seq data in zebrafish embryos [13]. We applied the SVM_DNaseI_ trained with the medaka dataset to the zebrafish genome. As the DNase I-seq data were not available for blastula-stage zebrafish embryos, we were unable to determine the position of accessible linker at zebrafish HMD boundaries like we did in medaka analyses. We therefore investigated the nucleosome pattern and SVM_DNaseI_ score only around the TSSs in HMDs and methylated regions. We found that in both medaka and zebrafish, nucleosome positions are phased and positioned around the TSSs that reside in HMDs, and that the SVM_DNaseI_ score was periodically high at linker regions (Fig. 5a, b left). By contrast, such periodicity was not observed for both nucleosome and SVM score around the methylated TSSs (Fig. 5a, b right). These results suggest that the specific 6-mers occurrence at nucleosome linkers in the HMD is conserved between the two distantly related teleost species.

## Discussion

Thus far, prediction of the nucleosome position on the basis of DNA sequence has not been successful in vertebrate genomes, in particular, gene promoter regions. In vertebrates, most gene promoters reside in HMDs, and in the present study, we reassessed the DNA sequence preference for nucleosome positioning in these specific regions. DNase I-seq was recently applied to genome-wide mapping of nucleosome positions in yeast and human [27], but in medaka embryos, DNase I was found to preferentially digest long linker DNA in HMDs. This feature allowed us to unveil the clear transition in nucleosome spacing length at the HMD boundary; from closed (180-bp interval in methylated) to open (200-bp in HMD) nucleosome organization. More importantly, with this precise map of linkers in HMDs, we identified the novel sequence-based rule that allows us to accurately predict the positions of nucleosomes in vertebrate HMDs harboring gene promoters. The 200-bp periodic occurrence of the predictable 6-mers accounts for longer spacing of nucleosomes in HMDs, and thereby promoters in HMDs could maintain accessibility to regulatory proteins (Fig. 4d). In general, the majority of hypomethylated promoters persist throughout cell differentiation and sustain gene expression of housekeeping genes and early developmental genes [7, 43, 44]. Thus, DNA sequence directed long nucleosome linkers could contribute to their transcriptional regulation by constitutively maintaining accessible nucleosome organizations at those promoters. On the other hand, cell-type specifically hypomethylated promoters may not depend on the predictable 6-mers identified in blastula embryos, as they are activated by cell-type-specific transcription factors and epigenetic modifications. Notably, this HMD-specific rule was at least conserved among teleosts, as the similar tendency was observed in zebrafish which is evolutionarily long diverged from medaka. However, this rule holds true only in HMDs, and the co-occurrence of the specific 6-mers and nucleosome linker is not observed in methylated regions. Since the HMD constitutes only 3% of the entire genome, despite its crucial role in gene regulation, the HMD-specific rule could have been overlooked in previous genome-wide analyses.

The strong phasing of nucleosome positions downstream of TSSs is widely conserved among eukaryote genomes, but the degree of sequence contribution to the nucleosome positioning varies among species [11, 13, 14]. Surprisingly, the novel HMD-specific rule in medaka clearly contradicts the global sequence preference previously reported (poly(dA:dT) for linkers and GC-rich for nucleosome cores) [11]; the predictable short sequences enriched in medaka HMD linkers are relatively GC-rich, and the Kaplan occupancy, which was originally used to predict the global nucleosome occupancy in yeast, exhibits the opposite tendency in HMDs. At the moment, the reason for the reverse sequence preference of nucleosomes and the function of high SVM_DNaseI_-weight 6-mers remain speculative. Those 6-mers may be intrinsically unfavorable for nucleosome formation in HMDs, although we cannot rule out the possibility that unknown proteins bind to those 6-mers and influence nucleosome positioning. To examine whether the 6-mers alone can direct nucleosome positioning, it would be informative to perform in vitro reconstitution of chromatin from histone octamers and naked medaka genomic DNA. Importantly, however, it has been reported in zebrafish that the strongly phased nucleosome array at gene promoters does not exist in early embryos, but appears during the zygotic genome activation (ZGA) stage, correlating with the emergence of H3K4me3, a histone modification specific to HMDs [13, 21, 45, 46]. This indicates that nucleosome positioning in promoter regions is not solely determined by DNA sequence, but may require specific chromatin environment (e.g., modifications such as H3K4me3 or binding of chromatin factors which function at the ZGA stage). Therefore, it is likely that the specific epigenetic environment override the normal sequence preference of nucleosome in HMDs.

## Conclusion

In summary, although the molecular mechanisms by which identified short sequences are translated into nucleosome positioning remain elusive, the present study focusing on the HMD provides novel insights into a hypomethylated DNA-specific regulation of nucleosome positioning in the vertebrate genome.

## Materials and methods

### Fish strains

We used medaka d-rR strain as wild type. Medaka fishes were maintained and raised under standard condition.

### DNase I-seq

DNase I-seq was performed as previously described [47] with modifications. 5000 d-rR strain medaka blastula embryos were dechorionated and dissociated by forcing the embryos through a 21G needle using a syringe, and cells were harvested by centrifugation at 500*g* for 5 min. After washing with PBS, cells were resuspended in 500 μl of buffer A [15 mM Tris–HCl (pH 8.0), 15 mM NaCl, 60 mM KCl, 1 mM EDTA, 0.5 mM EGTA, 1 mM PMSF]. Cells were isolated using a cell-strainer (Falcon, 352235), centrifuged at 500*g* for 10 min, and resuspended in 1.5 ml of lysis buffer [buffer A with 0.1% IGEPAL CA-630]. After a 1-min incubation at 4 °C, nuclei were collected by centrifugation at 500*g* for 10 min. Nuclei were washed in buffer A, then resuspended in nuclear storage buffer [20 mM Tris–HCl pH 8.0, 75 mM NaCl, 0.5 mM EDTA, 50% (v/v) glycerol, 1 mM DTT, and 0.1 mM PMSF], and stored at −80 °C. For DNase I digestion, frozen nuclei were thawed on ice, washed in buffer A with 0.5 mM spermidine and 0.3 mM spermine, incubated for exactly 2 min at 37 °C in 3.5 ml of buffer D [1 volume of 10× DNase I digestion buffer with 9 volume of Buffer A] containing 480 U of DNase I. The reaction was stopped by adding stop buffer [50 mM Tris–HCl, pH 8.0, 100 mM NaCl, 0.1% SDS, 100 mM EDTA, 20 μg/ml RNase A, 0.5 mM spermidine, and 0.3 mM spermine] and proteinase K, and incubated at 55 °C overnight. Digested DNA was purified by phenol chloroform, sucrose fractionated, and fragments below 1 kb were collected, end-repaired, ligated with adapters compatible with the Illumina sequencing platform and sequenced as single-end tags on HiSeq 1500 platform (Illumina).

### ATAC-seq

ATAC-seq was performed as previously described [37] with some modifications. Embryos were homogenized in PBS, and cells were harvested by centrifugation at 500*g* for 5 min. Approximately 5000 cells were used. After washing with PBS, cells were resuspended in 500 μl of cold lysis buffer [10 mM Tris–HCl pH7.4, 10 mM NaCl, 3 mM MgCl_2_, 0.1% Igepal CA-630], centrifuged for 10 min at 500*g*, and supernatant was removed. Tagmentation reaction was performed as described previously [37] with Nextera Sample Preparation Kit (Illumina). After DNA was purified using MinElute kit (Qiagen), two sequential PCR were performed to enrich small DNA fragments. First, 9-cycle PCR were performed using indexed primers from Nextera Index Kit (Illumina) and KAPA HiFi HS ReadyMix, and amplified DNA was size selected (less than 500 bp) using AMPure XP beads. Then, a second 7-cycle PCR were performed using the same primer as the first PCR, and purified by AMPure XP beads. Libraries were sequenced using the Illumina HiSeq 1500 platform.

### DNase I-seq and ATAC-seq data processing

The sequenced tags were aligned to the medaka reference genome by BWA [48], and tags with mapping quality larger than 20 were used for further analyses. Before the peak detection, each read position was shifted toward 5′ side with 50 bp. Then, MACS2 (version 2.0.10.20120913) [29] was used to identify regions that are significantly enriched (FDR 0.1%, fold enrichment > 5) with sequence tags (DHSs) using following options: –keep-dup all –nomodel –shiftsize 50 –q 0.01 –nolambda –call-summits –B –SPMR. For visualization and further analyses, signals per million reads data produced by MACS2 were used.

### Nucleosome organization and sequence profiles around HMD boundaries

We used HMDs identified in the previous study [23]. To calculate the average chromatin profile around HMD boundaries, we needed to set the base position (position *x* = 0) in each HMD. For this, we first determined DNase I-seq peak summits that locate within HMDs and selected the summit nearest to the boundary (the first low-methylated CpG in the HMD) as the base position. Then, the boundaries were classified by the orientation relative to the direction of transcription from the nearest TSS. HMDs that have TSSs within 1 kb distance were used for this analysis. Kaplan nucleosome occupancy was calculated using the model previously reported [11]. SNP rate was calculated using the genome sequences of two medaka species, Hd-rR and HNI. SNPs identified in previous study [41] were used.

To estimate the average spacing of nucleosomes, we calculated the autocorrelation of nucleosome dyad score using acf function of R. The autocorrelation in HMDs and methylated regions were calculated using the average nucleosome dyad score (Fig. 1c, middle) at position *x* = 0, …, 1000 and *x* = −1000, …, −100, respectively, where *x* = 0 is the position of boundary DNase I-seq summit.

### SVM for nucleosome linker and HMD prediction

We used the previously described method [33] for *k*-mer SVM. For training of SVM_DNaseI_ we first selected DNase I-seq peak summits within HMDs as the center of accessible nucleosome linkers. Then, we selected the center of two adjacent DNase I-seq peak summits as the nucleosome core position if the distance between the two summits was longer than 150 bp. From the linker (positive) and the core (negative) regions, 100-bp sequences were extracted. The sequences not on chromosome 8 were used for training, and those on chromosome 8 were used as test data to draw ROC and precision–recall curve. To test the performance of the prediction of DNase I pattern, we calculated the SVM score for each of the HMDs in chromosome 8 with sliding window of 100 bp with a step of 20 bp. Then, at each step, the average of overlapping windows was calculated. The correlation between the average SVM score and DNase I signal level was calculated for each HMDs.

For the positive data set of SVM_DNA_, all HMD sequences below 3 kb were used. For the negative data set, ten copies of original HMD genome-coordinate set were randomly distributed on methylated regions using bedtools. HMD sequences and methylated sequences not on chromosome 8 were used for training, and those on chromosome 8 were used as test data (for Fig. 4b, c).

### Motif analyses

TOMTOM [49] was used to search motifs similar to 6-mers. JASPAR Vertebrates and UniPROBE Mouse databases were used as target motifs.

## Additional files

**Additional file 1.** The comparison between DNase I-seq pattern and histone ChIP-seq pattern. Average profiles of DNase I-seq signal (black), DNA methylation (orange), and H3K27ac ChIP-seq signal (blue) around the accessible nucleosome linkers at the HMD boundaries. Vertical green dashed lines indicate the position of nucleosome core estimated from MNase-seq data (see Fig. 1c). The top schema shows the position of nucleosomes (green ovals) and methylated CpGs (orange circle).

**Additional file 2.** The average nucleosome spacing in HMDs and methylated regions. The autocorrelation in HMDs and methylated regions were calculated using the average nucleosome dyad score (Fig. 1c, middle) for both 5′ (left) and 3′ (right) boundary regions.

**Additional file 3.** The performance of *k*-mer SVM for different *k*-mer length. ROC curve and the area under the ROC curve (auc) are shown for different *k*-mer length (*k* = 2, 3, 4, 5, 6, 7, 8).

**Additional file 4.** Validation of the performance of SVM_DNaseI_ by ATAC-seq. (A) An example of prediction of nucleosome linkers (DNase I accessible regions) by SVM_DNaseI_ in HMDs on chromosome 8. Dark purple indicates the score higher than 0, light purple, lower than 0. Pearson’s correlation and its *P* value between ATAC-seq signal and SVM_DNaseI_ score for every 20 bp along the HMD are shown on the right. (B) A histogram of correlations between ATAC-seq signal and SVM_DNaseI_ score for all HMDs on chromosome 8. Blue and gray boxes represent the number of HMDs with and without significant correlation (*P* < 0.05), respectively.

**Additional file 5.** SVM_DNaseI_-weights for all 6-mers. All 6-mers are listed with SVM-weight.

**Additional file 6.** Known TF binding motifs similar to top 20 SVM_DNaseI_ 6-mers. Top 20 6-mers are listed with known TF motifs.
