# Supplementary figures and images for: Hypomethylated domain-enriched DNA motifs prepattern the accessible nucleosome organization in teleosts
Source: Epigenetics Chromatin. 2017 Sep 20;10:44. doi: 10.1186/s13072-017-0152-2 (PMC5607494; doi:10.1186/s13072-017-0152-2)

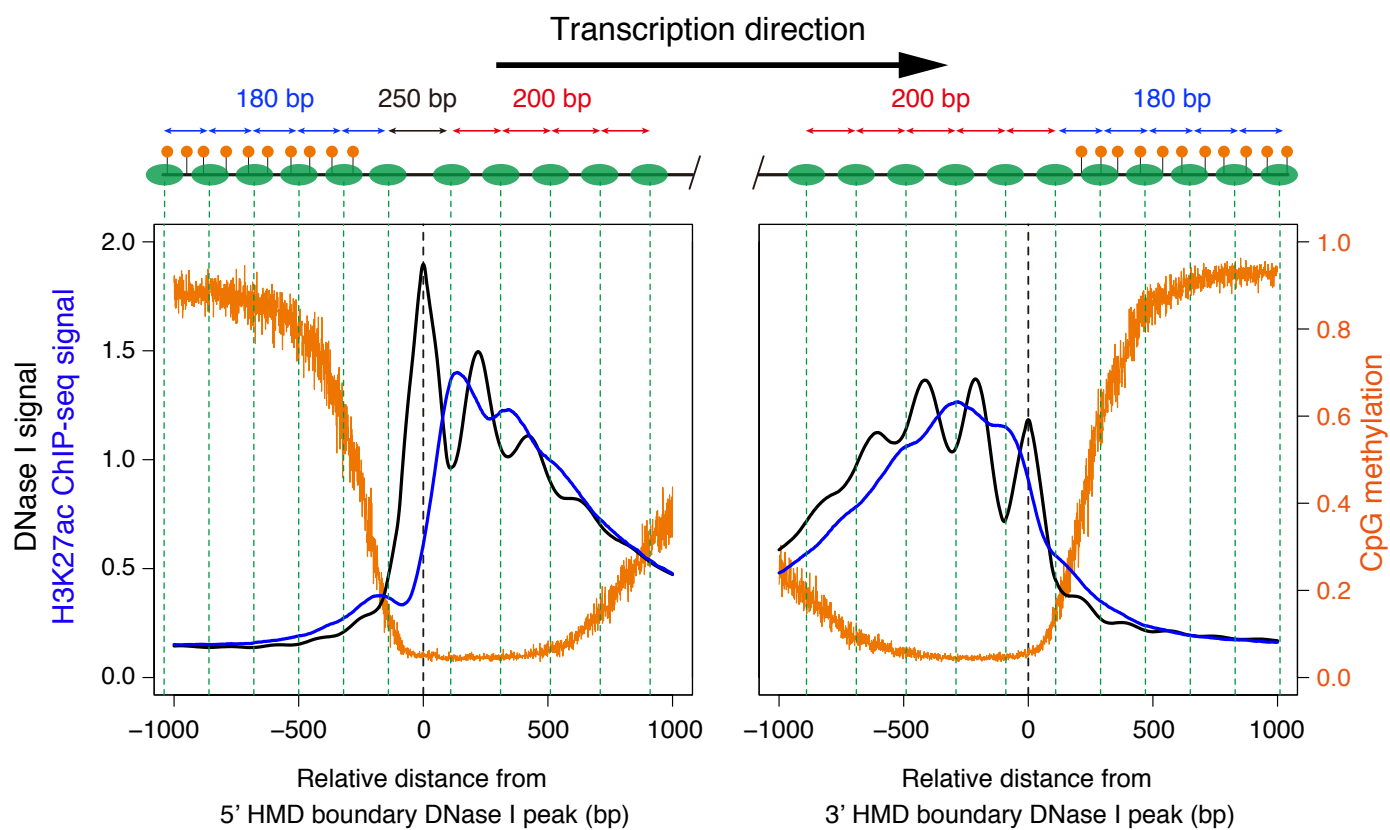

Supplement: Supplementary file 1 — Additional file 1. The comparison between DNase I-seq pattern and histone ChIP-seq pattern. Average profiles of DNase I-seq signal (black), DNA methylation (orange), and H3K27ac ChIP-seq signal (blue) around the accessible nucleosome linkers at the HMD boundaries. Vertical green dashed lines indicate the position of nucleosome core estimated from MNase-seq data (see Fig. 1c). The top schema shows the position of nucleosomes (green ovals) and methylated CpGs (orange circle). [file 13072_2017_152_MOESM1_ESM.pdf]

5' boundary (inside HMD)

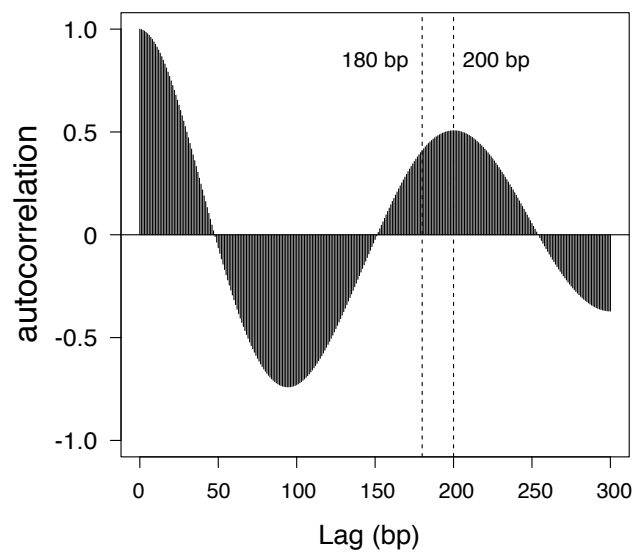

3' boundary (inside HMD)

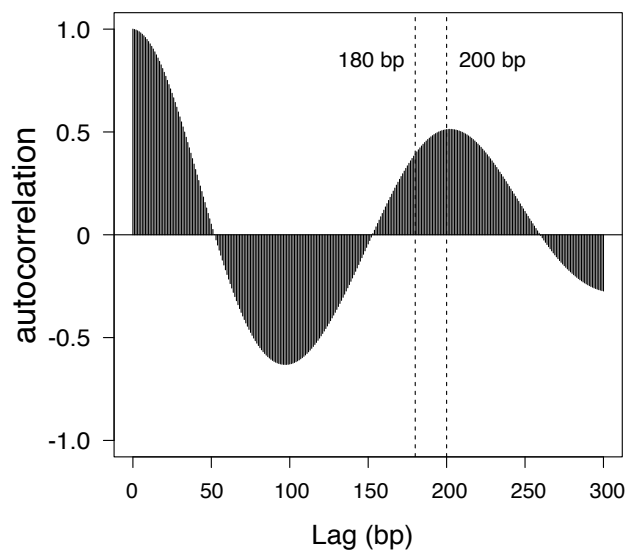

5' boundary (outside HMD)

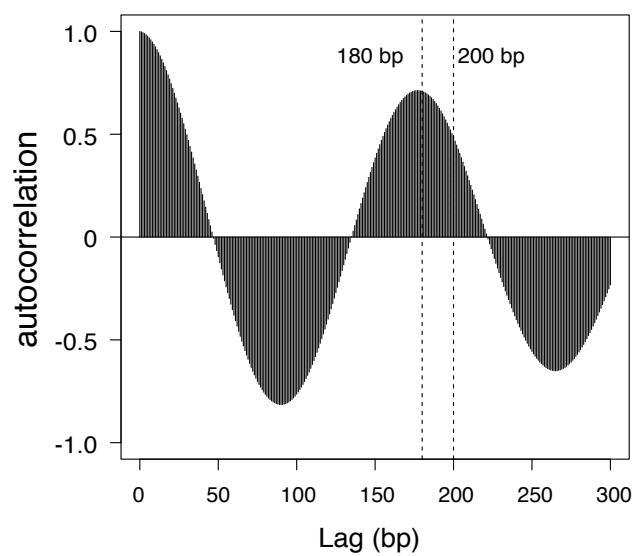

3' boundary (outside HMD)

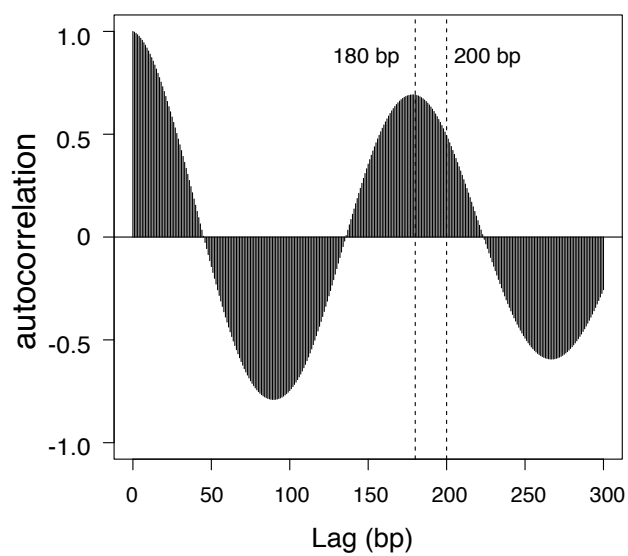

Supplement: Supplementary file 2 — Additional file 2. The average nucleosome spacing in HMDs and methylated regions. The autocorrelation in HMDs and methylated regions were calculated using the average nucleosome dyad score (Fig. 1c, middle) for both 5′ (left) and 3′ (right) boundary regions. [file 13072_2017_152_MOESM2_ESM.pdf]

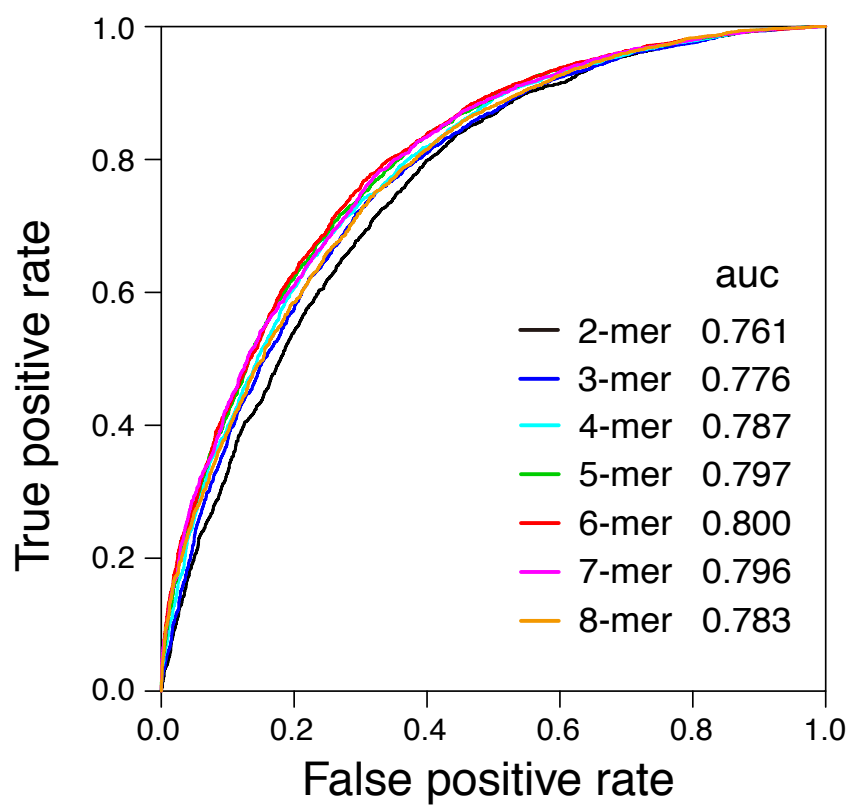

Supplement: Supplementary file 3 — Additional file 3. The performance of k-mer SVM for different k-mer length. ROC curve and the area under the ROC curve (auc) are shown for different k-mer length (k = 2, 3, 4, 5, 6, 7, 8). [file 13072_2017_152_MOESM3_ESM.pdf]

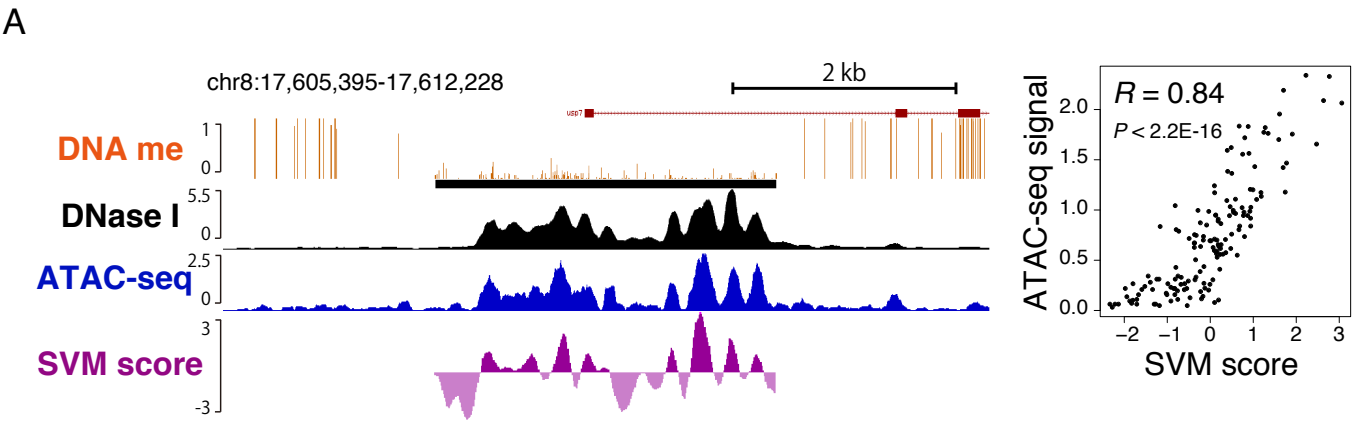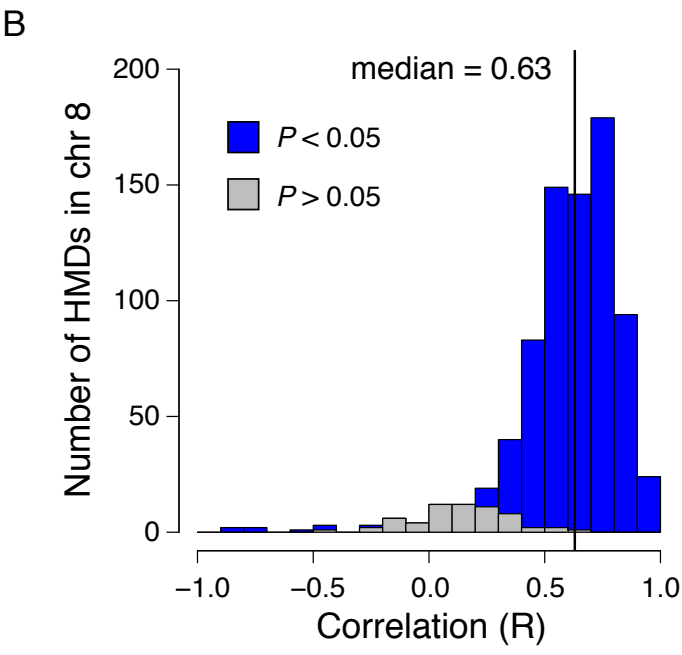

Supplement: Supplementary file 4 — Additional file 4. Validation of the performance of SVMDNaseI by ATAC-seq. (A) An example of prediction of nucleosome linkers (DNase I accessible regions) by SVMDNaseI in HMDs on chromosome 8. Dark purple indicates the score higher than 0, light purple, lower than 0. Pearson’s correlation and its P value between ATAC-seq signal and SVMDNaseI score for every 20 bp along the HMD are shown on the right. (B) A histogram of correlations between ATAC-seq signal and SVMDNaseI score for all HMDs on chromosome 8. Blue and gray boxes represent the number of HMDs with and without significant correlation (P < 0.05), respectively. [file 13072_2017_152_MOESM4_ESM.pdf]
